# A prognostic nomogram integrating preoperative IBI and MAR with clinicopathological factors for gastric cancer patients after radical gastrectomy

**DOI:** 10.3389/fsurg.2026.1793710

**Published:** 2026-03-20

**Authors:** Dong-Shan Sun, Yi-Fan Kong, Zheng-Yu Li, Hai-Xia Shan

**Affiliations:** Department of Oncology, The Affiliated Hospital of Xuzhou Medical University, Xuzhou, Jiangsu, China

**Keywords:** IBI, MAR, nomogram, overall survival, radical gastrectomy

## Abstract

**Objective:**

This study aimed to identify prognostic factors incorporating preoperative IBI and MAR levels and to construct a corresponding nomogram for patients undergoing radical gastrectomy for gastric cancer.

**Methods:**

We retrospectively analyzed 300 gastric cancer patients who underwent radical gastrectomy (June 2018–July 2021). Optimal cut-off values for preoperative IBI and MAR in predicting overall survival (OS) were determined by ROC analysis. Patients were stratified accordingly, and group differences were compared. OS was analyzed using Kaplan–Meier and log-rank tests. Independent clinicopathological prognostic factors were identified by Cox regression and used to build a nomogram predicting 1-, 3-, and 5-year survival. The model was internally validated via Bootstrap resampling and evaluated using the C-index, time-dependent ROC curves, and calibration plots.

**Results:**

The optimal cut-off values for IBI and MAR were 9.045 and 10.151, respectively. High IBI or MAR was associated with aggressive tumor features (all *P* < 0.05). Multivariate analysis identified adjuvant therapy, N stage, CA19-9, IBI, and MAR as independent prognostic factors. The resulting nomogram showed good discrimination, with C-indices of 0.809 (training) and 0.802 (validation). The AUCs for 1-, 3-, and 5-year OS all exceeded 0.83, and calibration was accurate. The nomogram successfully stratified patients into low- and high-risk groups with significantly different survival (Log-rank *P* < 0.001).

**Conclusion:**

Preoperative IBI and MAR are robust prognostic indicators in gastric cancer. The developed nomogram provides a practical visual tool for individualized postoperative risk assessment and management.

## 1 Introduction

Gastric cancer (GC) is a major digestive malignancy and a leading global health threat, ranking fourth in cancer mortality with over one million new cases annually (1). In China, GC remains a predominant cause of cancer-related morbidity and mortality (2, 3). Current management centers on surgical resection, supplemented by radiotherapy and chemotherapy. For early- to mid-stage disease (stages I–III), radical gastrectomy is the cornerstone curative intervention (4). Despite this, patients often face a poor prognosis, short survival, and high recurrence rates, posing a significant therapeutic challenge (5). Therefore, accurate assessment of disease status and timely adjustment of treatment are imperative to improve overall survival.

The inflammatory response and immune dysfunction are central to the initiation, progression, and metastasis of gastric cancer (GC), making them key targets for prognostic assessment (6, 7). In this context, the inflammatory burden index (IBI) has been developed as a novel multidimensional index. It quantifies the host-tumor inflammatory-immune interplay by integrating peripheral blood levels of C-reactive protein (CRP), neutrophils, and lymphocytes. Elevated IBI, indicative of excessive inflammation and impaired immunity, is associated with adverse survival outcomes (8, 9). Similarly, the monocyte-to-albumin ratio (MAR) serves as a composite marker of both inflammation and nutritional status. Emerging as a systemic inflammatory biomarker, MAR holds potential for assessing inflammatory status, nutritional level, and survival across various diseases, including cancer (10, 11).

While single inflammatory or nutritional markers offer limited prognostic value, composite indicators—such as the CRP-lymphocyte ratio and prognostic nutritional index (PNI)—hold greater promise for tumor prognosis modeling due to their multidimensional assessment capabilities (12, 13). Nevertheless, existing composites often focus exclusively on either the inflammation-immune axis or the nutrition-inflammation interface, failing to address the three core facets of tumor progression: immunity, inflammation, and nutrition. In contrast, the inflammatory burden index (IBI) and monocyte-to-albumin ratio (MAR) specifically target the inflammation-immune balance and inflammation-nutrition status, respectively. Their combined use could therefore provide a more comprehensive reflection of the patient's pathophysiological state. Currently, there is scant research on the combined prognostic value of preoperative IBI and MAR for long-term overall survival (OS) after radical gastrectomy. To address this gap, we developed and validated a clinically applicable nomogram incorporating these indices to inform personalized postoperative strategies and risk stratification.

## 2 Materials and methods

### 2.1 Patient characteristics

This retrospective cohort study enrolled gastric cancer patients who underwent radical gastrectomy at the Affiliated Hospital of Xuzhou Medical University between June 2018 and July 2021. Patients were included if they met all of the following criteria: (1) underwent radical gastrectomy for gastric cancer; (2) had a postoperative pathological confirmation of primary gastric cancer; (3) received no neoadjuvant radiotherapy or chemotherapy; (4) had no distant metastasis (M0 stage); and (5) had complete follow-up data. Conversely, exclusion criteria comprised: (1) concomitant malignancy in other organs or systems; (2) recent (within one month prior to surgery) or ongoing anti-inflammatory or immunosuppressive therapy; (3) presence of distant metastasis (M1 stage); (4) active infection, autoimmune disease, or hematologic disorder that could confound inflammatory marker measurement; (5) severe cardiac, hepatic, or renal dysfunction; and (6) incomplete follow-up. The study protocol was approved by the Ethics Committee of the Affiliated Hospital of Xuzhou Medical University (approval no. XYFY2025-KL640-01) and conducted in accordance with the Declaration of Helsinki. The requirement for written informed consent was waived due to the retrospective design.

### 2.2 Data collection

Clinical data were retrospectively extracted from the electronic medical record system. These included: (1) baseline characteristics (age, gender, body mass index); (2) laboratory indicators—C-reactive protein, neutrophil, lymphocyte, and monocyte counts, serum albumin, carcinoembryonic antigen (CEA), and carbohydrate antigen 19-9 (CA19-9); (3) histopathological features—tumor location, size, histological grade, T and N stages, Ki-67 index, and presence of perineural or lymphovascular invasion; and (4) postoperative adjuvant therapy status. To ensure data quality and minimize bias, two independent researchers performed the data extraction and assessment. Both reviewers were blinded to the study's outcome data during this process.

### 2.3 IBI and MAR definitions

Fasting peripheral venous blood samples were collected from all patients within 7 days before surgery. Levels of C-reactive protein (CRP), neutrophils, lymphocytes, monocytes, and serum albumin were measured using a Sysmex XE-2100 automatic hematology analyzer with manufacturer-supplied reagents. The inflammatory burden index (IBI) and monocyte-to-albumin ratio (MAR) were then calculated using the following formulas: IBI = [CRP (mg/L) × absolute neutrophil count (*10⁹/L*)]/absolute lymphocyte count (*10⁹/L*); MAR = [absolute monocyte count (*10⁹/L*) × 10^3^]/serum albumin (g/L). Calculations were performed using raw laboratory values without additional data standardization or preprocessing to maintain clinical applicability. To ensure accuracy and consistency, all laboratory procedures were strictly controlled. Qualified personnel performed the assays in accordance with standardized operating procedures (SOPs). Equipment was regularly calibrated, and any anomalous results were rechecked prior to analysis.

### 2.4 Follow-up

Follow-up was conducted via medical record review and telephone interviews according to a predefined schedule: initial assessment at 3 months after surgery, then every 3 months for the first 2 years, and every 6 months thereafter until year 5. All patients were followed until death or June 30, 2025, whichever occurred first. Overall survival (OS) was defined as the time from the date of surgery to death from any cause or to the last follow-up.

### 2.5 Statistical analysis

All statistical analyses were performed using R software (version 4.4.1). Missing data were handled using complete-case analysis. Patients with incomplete clinical parameters or follow-up information (*n* = 177) were excluded from the final cohort prior to analysis, consistent with the strict inclusion and exclusion criteria. The entire cohort was randomly split into a training set (*n* = 210) and an internal validation set (*n* = 90) at a 7:3 ratio for model development and validation, respectively, with the random seed set to 123. Continuous variables were assessed for normality using the Kolmogorov–Smirnov test. Normally distributed data are expressed as mean ± standard deviation and compared with the independent samples *t*-test; non-normally distributed data are presented as median (interquartile range) and compared with the Mann–Whitney *U*-test. Categorical variables are expressed as number (percentage) and compared using the chi-square test or Fisher's exact test, as appropriate.

The primary endpoint was overall survival (OS), defined as the time from surgery to death from any cause. Patients alive at the last follow-up (June 30, 2025) were censored. Survival probabilities were estimated using the Kaplan–Meier method and compared between groups with the log-rank test. The predictive efficacy of preoperative IBI and MAR was evaluated using receiver operating characteristic (ROC) curve analysis, with optimal cut-off values determined by maximizing the Youden index for subsequent patient stratification.

In the training set, potential prognostic factors for OS were first screened by univariate Cox proportional hazards regression. Variables with *P* < 0.05 were then entered into a multivariate Cox regression model using the Enter method to identify independent predictors. Before finalizing the model, the proportional hazards assumption was verified using Schoenfeld residuals, and no violations were observed (*P* > 0.05 for all variables). Additionally, multicollinearity was assessed using the Variance Inflation Factor (VIF), with all VIF values < 5, indicating the absence of significant collinearity among the independent predictors. Based on these independent factors, a nomogram was constructed to predict 1-, 3-, and 5-year overall survival probabilities. The model's performance was comprehensively evaluated. Discrimination was assessed using the concordance index (C-index) and time-dependent ROC curves. Calibration was evaluated by plotting predicted against observed survival probabilities. Internal validation was performed using bootstrap resampling with 1,000 iterations to correct for optimism. The procedure involved: (1) calculating the apparent performance (C-index, time-dependent AUC, and calibration) in the original training set; (2) generating 1,000 bootstrap samples via resampling with replacement; (3) refitting the model on each bootstrap sample and evaluating it on both the bootstrap sample and the original training set to determine the optimism (performance difference); and (4) subtracting the average optimism from the apparent performance to derive optimism-corrected metrics.

For clinical risk stratification, the optimal cut-off value for the nomogram-derived linear predictor was determined in the training set using the “Surv-Cutpoint” function based on maximally selected rank statistics, dichotomizing patients into low- and high-risk groups. This cut-off was then applied to the validation set. Survival differences between the risk groups were compared using Kaplan–Meier curves and the log-rank test. All statistical tests were two-sided, and a *P*-value < 0.05 was considered statistically significant.

## 3 Results

### 3.1 Predictive performance of preoperative IBI and MAR for OS

**Figure 1:**
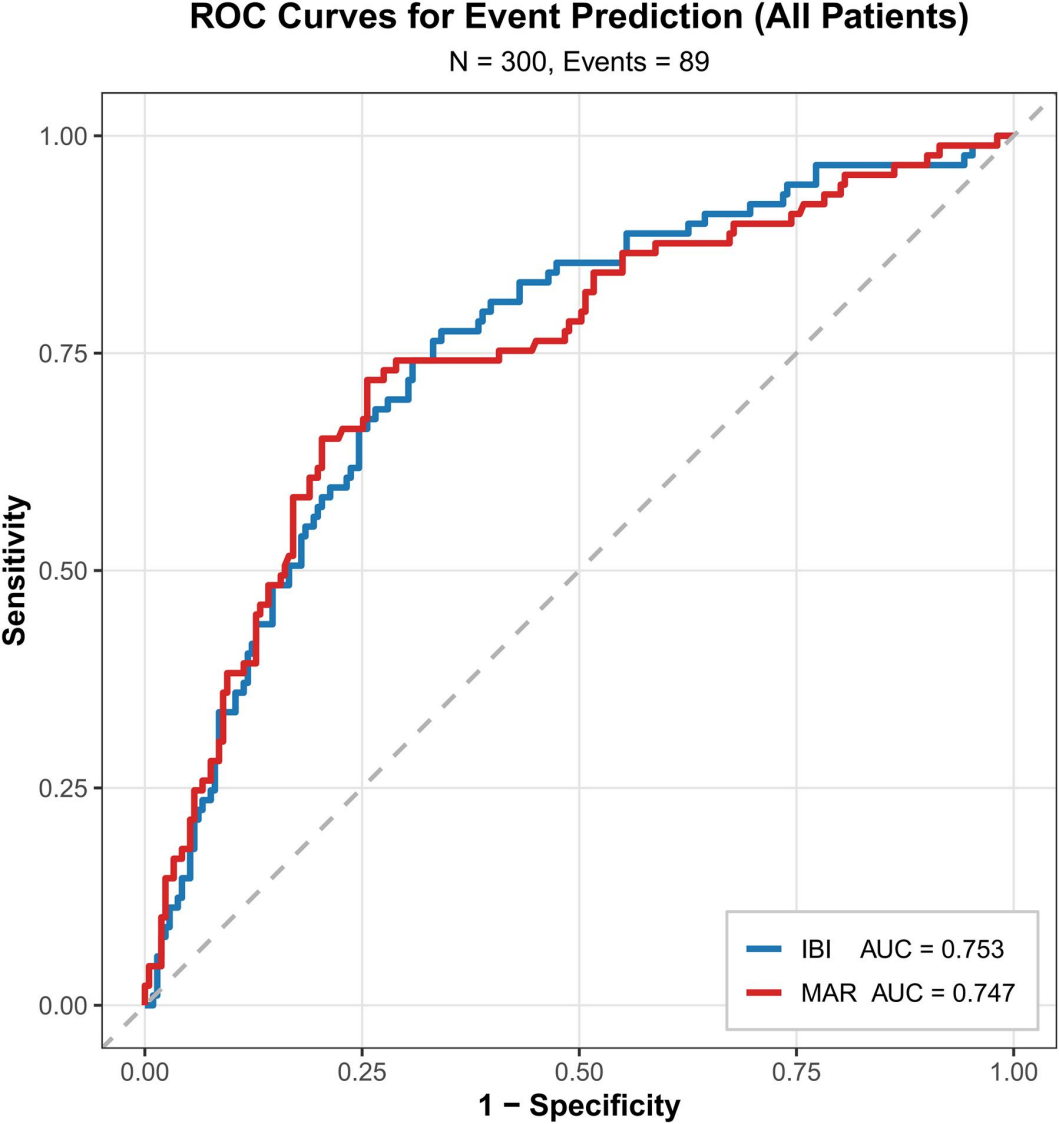
ROC curve of IBI in predicting overall survival (OS) of patients after radical gastrectomy for gastric cancer. IBI=[CRP (mg/L) × absolute neutrophil count (*10⁹/L*)]/absolute lymphocyte count (*10⁹/L*); MAR=[absolute monocyte count (*10⁹/L*) × 10^3^]/serum albumin (g/L); AUC, Area Under the Receiver Operating Characteristic (ROC) Curve.

As shown in Figure 1, the area under the curve (AUC) of preoperative IBI and MAR for predicting prognosis after radical gastrectomy were 0.753 (95% CI: 0.69–0.81) and 0.747 (95% CI: 0.68–0.81), respectively. The optimal cut-off values were 9.045 for IBI and 10.151 for MAR, with corresponding sensitivities of 77.53% and 71.91%, and specificities of 65.88% and 74.41%. The Youden index was selected to determine these thresholds to objectively maximize the summation of sensitivity and specificity. Given the absence of established clinical reference ranges for IBI and MAR in this specific population, this data-driven approach minimizes subjective bias and optimizes the discriminatory power of the biomarkers. Details are presented in Table 1**.**

**Table 1:** Comparison of the predictive efficacy of IBI and MAR for prognosis in 300 gastric cancer patients.

Variables	Cut-off value	Sensitivity/%	Specificity/%	AUC	95%CI	*P*-value
IBI	9.045	77.53	65.88	0.753	(0.69,0.81)	<0.001
MAR	10.151	71.91	74.41	0.747	(0.68,0.81)	<0.001

IBI=[CRP (mg/L) × absolute neutrophil count (*10⁹/L*)]/absolute lymphocyte count (*10⁹/L*); MAR=[absolute monocyte count (*10⁹/L*) × 10^3^]/serum albumin (g/L); AUC, Area Under the Receiver Operating Characteristic (ROC) Curve.

### 3.2 Clinicopathological characteristics by IBI and MAR groups

**Figure 2:**
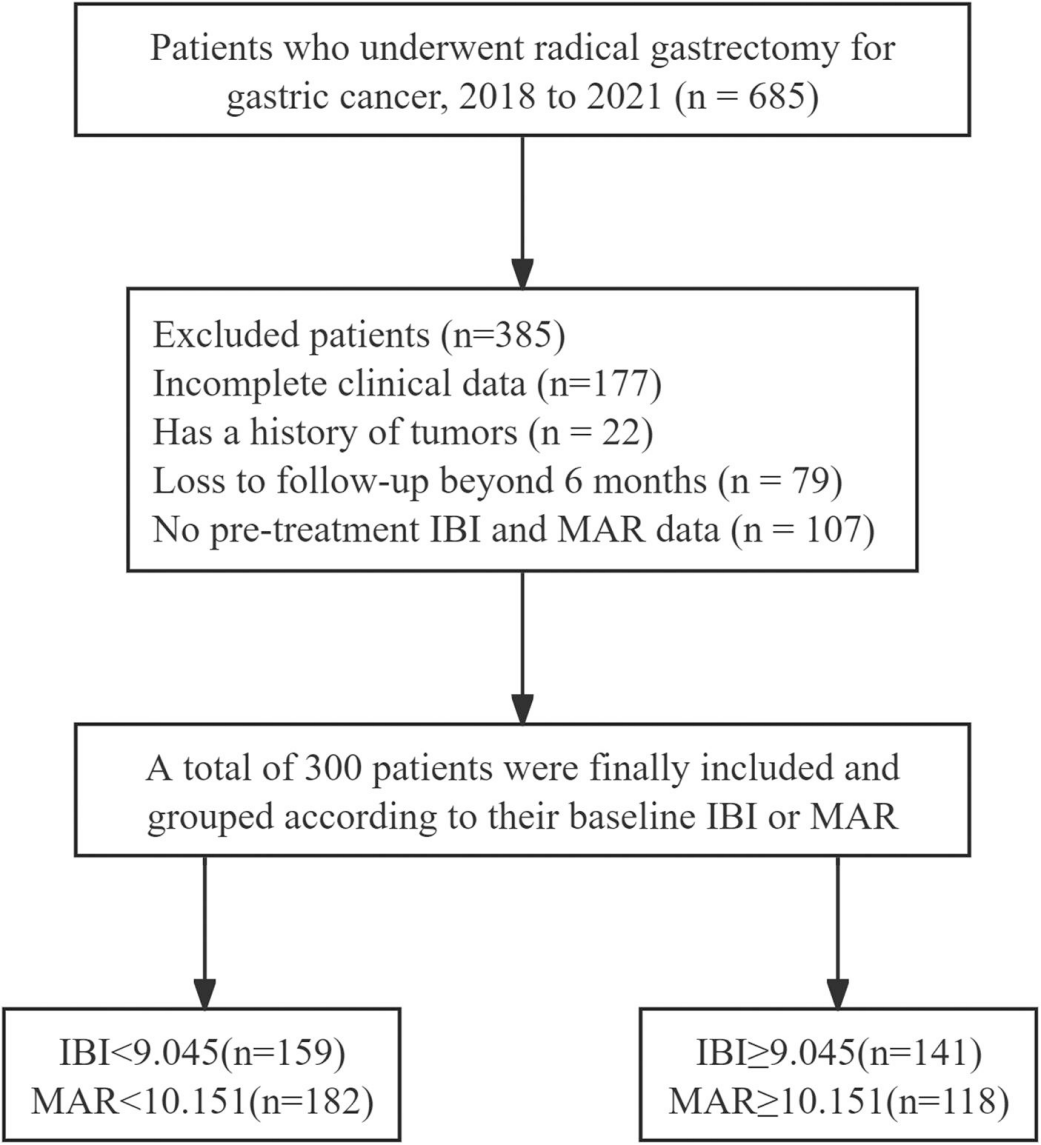
Flow chart illustrates the screening situation of patients with gastric cancer who have undergone radical gastrectomy. IBI: inflammatory burden index; MAR: monocyte-to-albumin ratio.

This retrospective study analyzed 300 gastric cancer patients who underwent radical gastrectomy between June 2018 and July 2021(see Figure 2). Using the optimal cut-off values, patients were stratified into low- and high-expression subgroups for IBI and MAR, respectively. Accordingly, the cohort was divided into a low IBI group (IBI < 9.045, *n* = 159) and a high IBI group (IBI ≥ 9.045, *n* = 141), as well as a low MAR group (MAR < 10.151, *n* = 182) and a high MAR group (MAR ≥ 10.151, *n* = 118). The detailed distribution of clinicopathological features across these groups is summarized in Table 2. However, no statistically significant differences were observed in demographic characteristics such as age and gender between the groups (all *P* > 0.05).

**Table 2:** Comparison of clinicopathological characteristics between different IBI and MAR groups/n (%).

Variable	IBI		*χ* ^2^	*P-*value	MAR		χ^2^	*P-*value
	IBI<9.045	IBI≥9.045			MAR<10.151	MAR≥10.151		
*N* = 300	*N* = 159	*N* = 141			*N* = 182	*N* = 118		
Gender			0.002	0.996			0.421	0.517
Female	47 (29.6)	42 (29.8)			57 (31.3)	32 (27.1)		
Male	112 (70.4)	99 (70.2)			125 (68.7)	86 (72.9)		
Differentiation			5.464	**0.019**			4.768	**0**.**029**
High/Middle	49 (30.8)	26 (18.4)			54 (29.7)	21 (17.8)		
Low	110 (69.2)	115 (81.6)			128 (70.3)	97 (82.2)		
Age			0.148	0.701			1.456	0.228
<60	62 (39.0)	51 (36.2)			74 (40.7)	39 (33.1)		
≥60	97 (61.0)	90 (63.8)			108 (59.3)	79 (66.9)		
BMI			1.364	0.243			0.078	0.780
<25	119 (74.8)	96 (68.1)			132 (72.5)	83 (70.3)		
≥25	40 (25.2)	45 (31.9)			50 (27.5)	35 (29.7)		
Adjuvant therapy			4.305	**0.038**			0.774	0.379
No	30 (18.9)	42 (29.8)			40 (22.0)	32 (27.1)		
Yes	129 (81.1)	99 (70.2)			142 (78.0)	86 (72.9)		
Location			4.788	0.091			1.803	0.406
Fundus	32 (20.1%)	35 (24.8%)			37 (20.3)	30 (25.4)		
Body	58 (36.5%)	35 (24.8%)			61 (33.5)	32 (27.1)		
Antrum	69 (43.4%)	71 (50.4%)			84 (46.2)	56 (47.5)		
Tumor longest diameter			7.035	**0.008**			5.887	**0**.**015**
<5cm	103 (64.8)	69 (48.9)			115 (63.2)	57 (48.3)		
≥5cm	56 (35.2)	72 (51.1)			67 (36.8)	61 (51.7)		
T stage			21.353	**<0.001**			13.786	**0**.**003**
T1	37 (23.3)	13 (9.2)			42 (23.1)	8 (6.8)		
T2	31 (19.5)	17 (12.1)			26 (14.3)	22 (18.6)		
T3	66 (41.5)	64 (45.4)			73 (40.1)	57 (48.3)		
T4	25 (15.7)	47 (33.3)			41 (22.5)	31 (26.3)		
N stage			18.046	**<0.001**			11.842	**0**.**008**
N0	70 (44.0)	30 (21.3)			73 (40.1)	27 (22.9)		
N1	26 (16.4)	28 (19.9)			34 (18.7)	20 (16.9)		
N2	23 (14.5)	27 (19.1)			25 (13.7)	25 (21.2)		
N3	40 (25.2)	56 (39.7)			50 (27.5)	46 (39.0)		
Vein inv			10.340	**0.001**			13.006	**<0**.**001**
No	81 (50.9)	45 (31.9)			92 (50.5)	34 (28.8)		
Yes	78 (49.1)	96 (68.1)			90 (49.5)	84 (71.2)		
Nerve inv			1.709	0.191			5.279	**0**.**022**
No	75 (47.2)	55 (39.0)			89 (48.9)	41 (34.7)		
Yes	84 (52.8)	86 (61.0)			93 (51.1)	77 (65.3)		
CEA			3.757	0.053			0.589	0.443
<5	128 (80.5)	99 (70.2)			141 (77.5)	86 (72.9)		
≥5	31 (19.5)	42 (29.8)			41 (22.5)	32 (27.1)		
CA19-9			5.792	**0.016**			6.548	**0**.**011**
<37	144 (90.6)	113 (80.1)			164 (90.1)	93 (78.8)		
≥37	15 (9.4)	28 (19.9)			18 (9.9)	25 (21.2)		
Ki-67			3.988	**0.046**			11.588	**0**.**001**
<50	55 (34.6)	33 (23.4)			67 (36.8%)	21 (17.8%)		
≥50	104 (65.4)	108 (76.6)			115 (63.2%)	97 (82.2%)		

Fundus, Fundus of stomach; Body, Body of stomach; Antrum, Antrum of stomach; T, tumor; N, Node; CEA, Carcinoembryonic antigen; CA19-9, Carbohydrate antigen 19-9.

Bold values indicate statistical significance (*P* < 0.05).

Patients in the high-expression groups for either IBI or MAR exhibited consistently more aggressive tumor characteristics. These included poorer differentiation, a larger tumor longest diameter, more advanced T and N stages, higher rates of vascular invasion, elevated CA19-9 levels, and increased Ki-67 expression (all *P* < 0.05). These findings indicate that elevated preoperative IBI and MAR are associated with a constellation of pathological features indicative of greater tumor invasiveness and adverse biology.

### 3.3 Baseline characteristics of the training and validation sets

To develop and validate the model, the total cohort (*N* = 300) was randomly split into a training set (*n* = 210) and a validation set (*n* = 90) at a 7:3 ratio. As shown in Table 3, the baseline clinicopathological characteristics—including gender, age, BMI, tumor location, tumor longest diameter, T/N stage, differentiation, vascular/nerve invasion, CEA, CA19-9, and Ki-67—were well-balanced between the two sets, with no statistically significant differences (all *P* > 0.05).

**Table 3:** Comparison of baseline characteristics between the training set and validation set/n (%).

Variables	Total [*n* = 300]	Train [*n* = 210]	Validation [*n* = 90]	χ^2^	*P*-value
Gender				0.779	0.377
Female	89 (29.7)	66 (31.4)	23 (25.6)		
Male	211 (70.3)	144 (68.6)	67 (74.4)		
Differentiation				1.354	0.244
Well/Moderately differentiated	75 (25.0)	57 (27.1)	18 (20.0)		
Poorly differentiated	225 (75.0)	153 (72.9)	72 (80.0)		
Age				0.782	0.377
<60	113 (37.7)	83 (39.5)	30 (33.3)		
≥60	187 (62.3)	127 (60.5)	60 (66.7)		
BMI				1.954	0.162
<25	215 (71.7)	145 (69.0)	70 (77.8)		
≥25	85 (28.3)	65 (31.0)	20 (22.2)		
Adjuvant therapy				0.014	0.910
No	72 (24.0)	50 (23.8)	22 (24.4)		
Yes	228 (76.0)	160 (76.2)	68 (75.6)		
Location				0.121	0.942
Fundus	67 (22.3%)	48 (22.9)	19 (21.1)		
Body	93 (31.0%)	65 (31.0)	28 (31.1)		
Antrum	140 (46.7%)	97 (46.2)	43 (47.8)		
Tumor longest diameter				0.053	0.819
<5cm	172 (57.3)	119 (56.7)	53 (58.9)		
≥5cm	128 (42.7)	91 (43.3)	37 (41.1)		
T stage				3.912	0.271
T1	50 (16.7)	38 (18.1)	12 (13.3)		
T2	48 (16.0)	34 (16.2)	14 (15.6)		
T3	130 (43.3)	94 (44.8)	36 (40.0)		
T4	72 (24.0)	44 (21.0)	28 (31.1)		
N stage				3.445	0.328
N0	100 (33.3)	70 (33.3)	30 (33.3)		
N1	54 (18.0)	41 (19.5)	13 (14.4)		
N2	50 (16.7)	30 (14.3)	20 (22.2)		
N3	96 (32.0)	69 (32.9)	27 (30.0)		
Vein inv				0.188	0.664
No	126 (42.0)	86 (41.0)	40 (44.4)		
Yes	174 (58.0)	124 (59.0)	50 (55.6)		
Nerve inv				0.145	0.703
No	130 (43.3)	93 (44.3)	37 (41.1)		
Yes	170 (56.7)	117 (55.7)	53 (58.9)		
CEA				0.497	0.481
<5	227 (75.7)	156 (74.3)	71 (78.9)		
≥5	73 (24.3)	54 (25.7)	19 (21.1)		
CA19-9				0.047	0.829
<37	257 (85.7)	181 (86.2)	76 (84.4)		
≥37	43 (14.3)	29 (13.8)	14 (15.6)		
Ki-67				1.165	0.28
<50	88 (29.3%)	66 (31.4)	22 (24.4)		
≥50	212 (70.7%)	144 (68.6)	68 (75.6)		

Fundus, Fundus of stomach; Body, Body of stomach; Antrum, Antrum of stomach; T, Tumor; N, Node; CEA, Carcinoembryonic antigen; CA19-9, Carbohydrate antigen 19-9.

### 3.4 Independent prognostic factors

In the training set (*n* = 210), potential prognostic factors for overall survival were initially screened using univariate Cox regression (Table 4). Tumor differentiation, adjuvant therapy, tumor longest diameter, T stage, N stage, vascular invasion, nerve invasion, CA19-9, and preoperative IBI and MAR levels were significantly associated with OS (all *P* < 0.05). These significant variables were then entered into a multivariate Cox model. After adjustment, adjuvant therapy, N stage, CA19-9, and preoperative IBI and MAR levels emerged as independent prognostic factors (all *P* < 0.05).

**Table 4:** Univariate and multivariate Cox regression analyses of factors affecting overall survival in the training Set.

Variables	Univariate		Multivariate	
	HR (95%CI)	*P* value	HR (95%CI)	*P* value
Gender				
Female	1.00 (Ref.)		-	-
Male	0.96 (0.56–1.64)	0.873	-	-
Differentiation				
Well/Moderately differentiated	1.00 (Ref.)		1.00 (Ref.)	
Poorly differentiated	2.47 (1.21–5.02)	0.013	1.57 (0.72–3.43,)	0.259
Age				
<60	1.00 (Ref.)		-	-
≥60	1.12 (0.66–1.90)	0.666	-	-
BMI				
<25	1.00 (Ref.)		-	-
≥25	1.29 (0.76–2.19)	0.346	-	-
Adjuvant therapy				
No	1.00 (Ref.)		1.00 (Ref.)	
Yes	0.51 (0.30–0.87)	0.013	0.20 (0.10–0.40)	**< 0.001**
Location				
Fundus	1.00 (Ref.)		-	**-**
Body	0.88 (0.45–1.73)	0.712	-	**-**
Antrum	0.83 (0.44–1.57)	0.570	-	-
Tumor longest diameter				
<5cm	1.00 (Ref.)		1.00 (Ref.)	
≥5cm	1.73 (1.04–2.87)	0.035	0.88 (0.48–1.62)	0.680
T stage				
T1	1.00 (Ref.)		1.00 (Ref.)	
T2	2.66 (0.82–8.65)	0.103	1.68 (0.47–6.03)	0.426
T3	3.38 (1.19–9.62)	0.022	1.32 (0.33–5.27)	0.692
T4	4.60 (1.56–13.59)	0.006	0.82 (0.18–3.76)	0.801
N stage				
N0	1.00 (Ref.)		1.00 (Ref.)	
N1	2.85 (1.01–8.01)	0.047	3.51 (0.97–12.72)	0.056
N2	5.38 (2.02–14.36)	<0.001	4.56 (1.23–16.84)	**0.023**
N3	6.93 (2.90–16.54)	<0.001	5.39 (1.50–19.35)	**0.010**
Vein inv				
No	1.00 (Ref.)		1.00 (Ref.)	
Yes	4.02 (2.04–7.93)	<0.001	1.07 (0.41–2.82)	0.892
Nerve inv				
No	1.00 (Ref.)		1.00 (Ref.)	
Yes	2.28 (1.30–4.00)	0.004	1.86 (0.84–4.14)	0.129
CEA				
<5	1.00 (Ref.)		-	-
≥5	1.51 (0.88–2.58)	0.134	-	-
CA19-9				
<37	1.00 (Ref.)		1.00 (Ref.)	
≥37	2.67 (1.49–4.79)	<0.001	1.95 (1.02–3.72)	**0.043**
Ki-67				
<50	1.00 (Ref.)		-	-
≥50	1.46 (0.81–2.62)	0.207	-	-
IBI	1.01 (1.01–1.02)	<0.001	1.01 (1.00–1.02)	**0.036**
MAR	1.19 (1.13–1.25)	<0.001	1.14 (1.08–1.21)	**<0.001**

HR, Hazards ratio; CI, Confidence interval; Ref., Reference; Fundus, Fundus of stomach; Body, Body of stomach; Antrum, Antrum of stomach; T, Tumor; N, Node; CEA, Carcinoembryonic antigen; CA19-9, Carbohydrate antigen 19-9; IBI and MAR were included in the analysis as continuous variables, with mean ± standard deviation values of 17.1 ± 25.0 and 9.5 ± 3.8, respectively, in the training set.

Bold values indicate statistical significance (*P* < 0.05).

### 3.5 Prognostic nomogram

**Figure 3:**
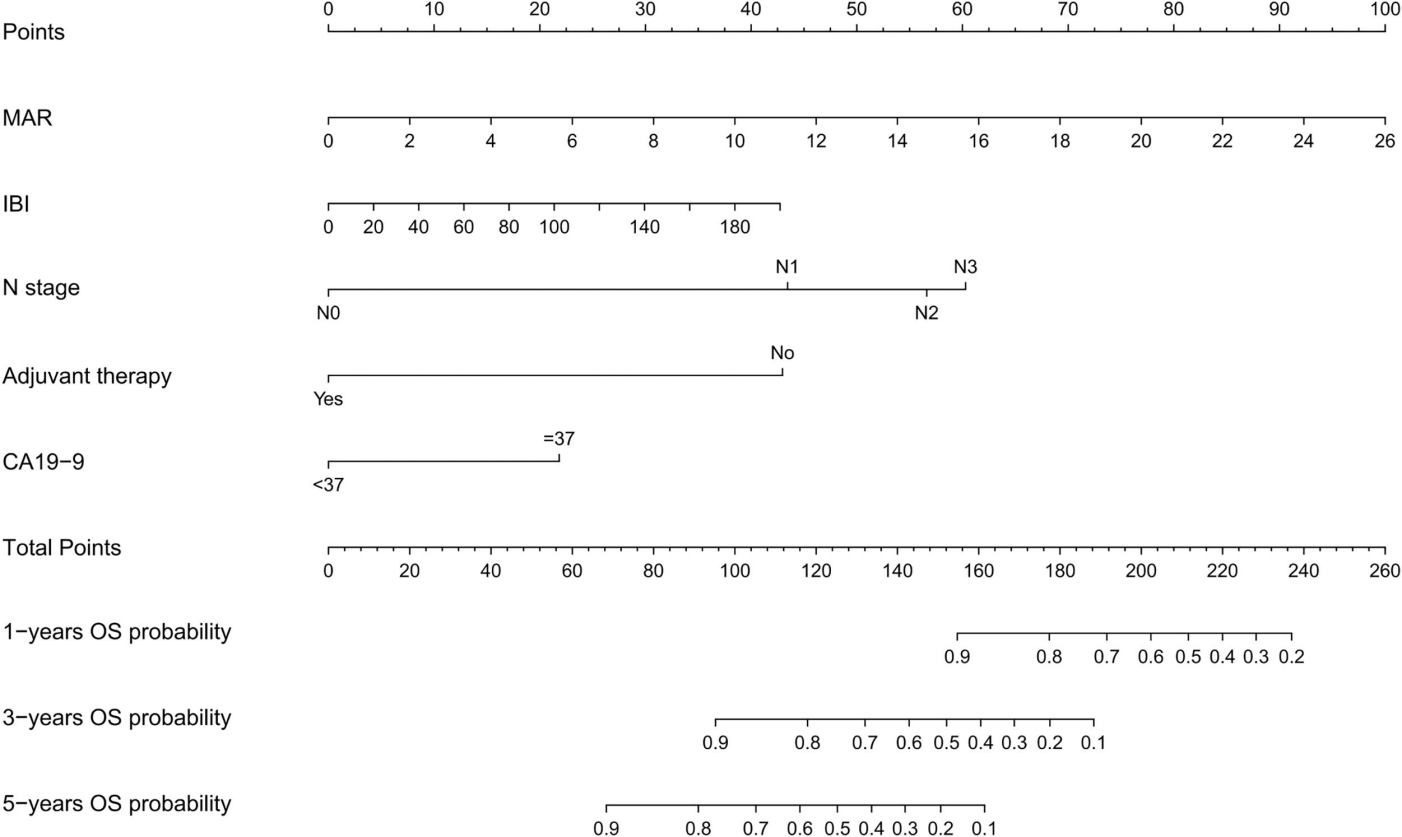
A nomogram for predicting 1-, 3-, and 5-year overall survival in patients after radical gastrectomy. IBI, Inflammatory burden index; MAR, Monocyte-to-albumin ratio; N, Node; CA19-9: Carbohydrate antigen 19-9; OS, Overall survival.

Based on the independent prognostic factors identified by multivariate Cox analysis (adjuvant therapy, N stage, CA19-9, preoperative IBI and MAR), a nomogram was developed using the rms package in R software (version 4.4.1) to predict 1-, 3-, and 5-year overall survival. The nomogram assigns points to each variable; summing these points yields a total score, which corresponds to a predicted survival probability on the lower axes of the chart **(**Figure 3).

### 3.6 Nomogram validation and performance

**Figure 4:**
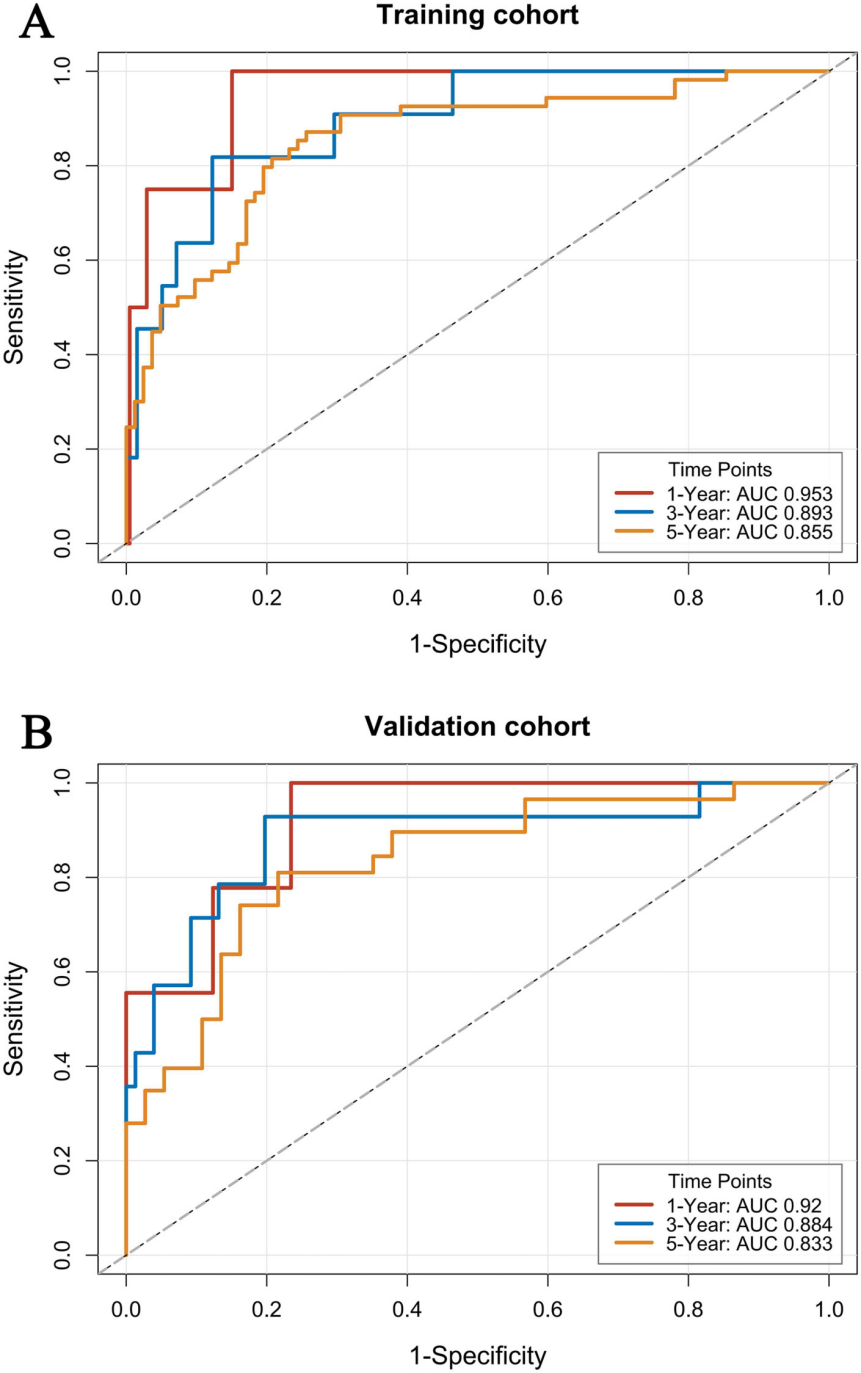
Time-dependent ROC curves of the nomogram for predicting 1-, 3-, and 5-year overall survival. **(A)** Training cohort; **(B)** Validation cohort; AUC, Area Under the Receiver Operating Characteristic (ROC) Curve.

**Figure 5:**
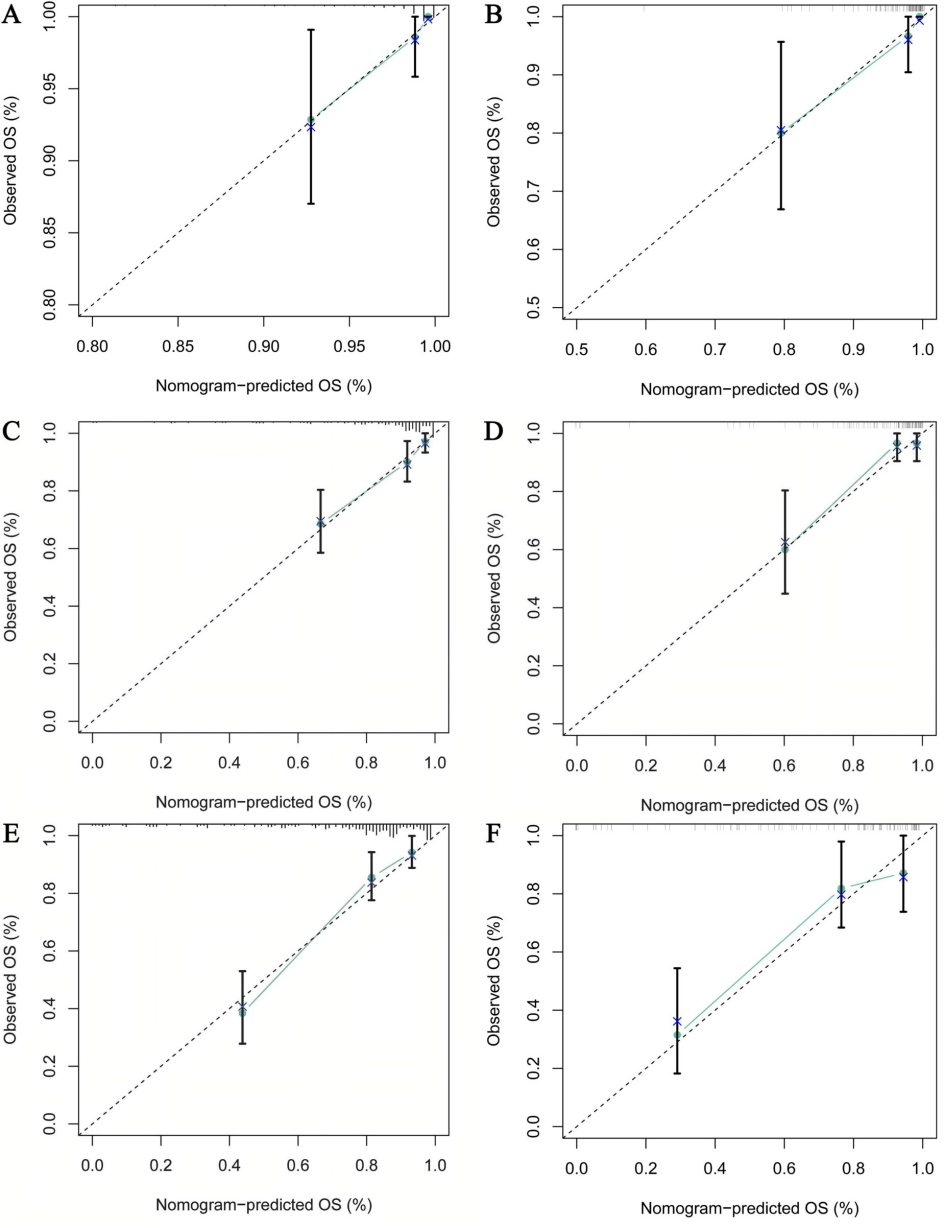
Calibration plots for the prognostic model. **(A)** Calibration plot for 1-year overall survival (OS) in the training set; **(B)** Calibration plot for 1-year OS in the validation set; **(C)** Calibration plot for 3-year OS in the training set; **(D)** Calibration plot for 3-year OS in the validation set; **(E)** Calibration plot for 5-year OS in the training set; **(F)** Calibration plot for 5-year OS in the validation set.

To evaluate model robustness, internal validation was performed in the training set using Bootstrap resampling with 1,000 repetitions. The nomogram demonstrated strong discrimination, with a concordance index (C-index) of 0.809 in the training set and 0.802 in the validation set, indicating stable performance and good reproducibility. Further time-dependent ROC analysis showed high AUCs for predicting 1-, 3-, and 5-year OS: 0.953, 0.893, and 0.855 in the training set **[**Figure 4**:** (A) Training cohort], and 0.920, 0.884, and 0.833 in the validation set [Figure 4**:** (B) Validation cohort]. The calibration curves for 1-, 3-, and 5-year OS in both the training and validation sets are shown in Figure 5**.** The close alignment between the nomogram-predicted probabilities and the observed outcomes along the 45° diagonal demonstrates good calibration, confirming the model's reliable predictive accuracy.

### 3.7 Risk stratification based on the nomogram

**Figure 6:**
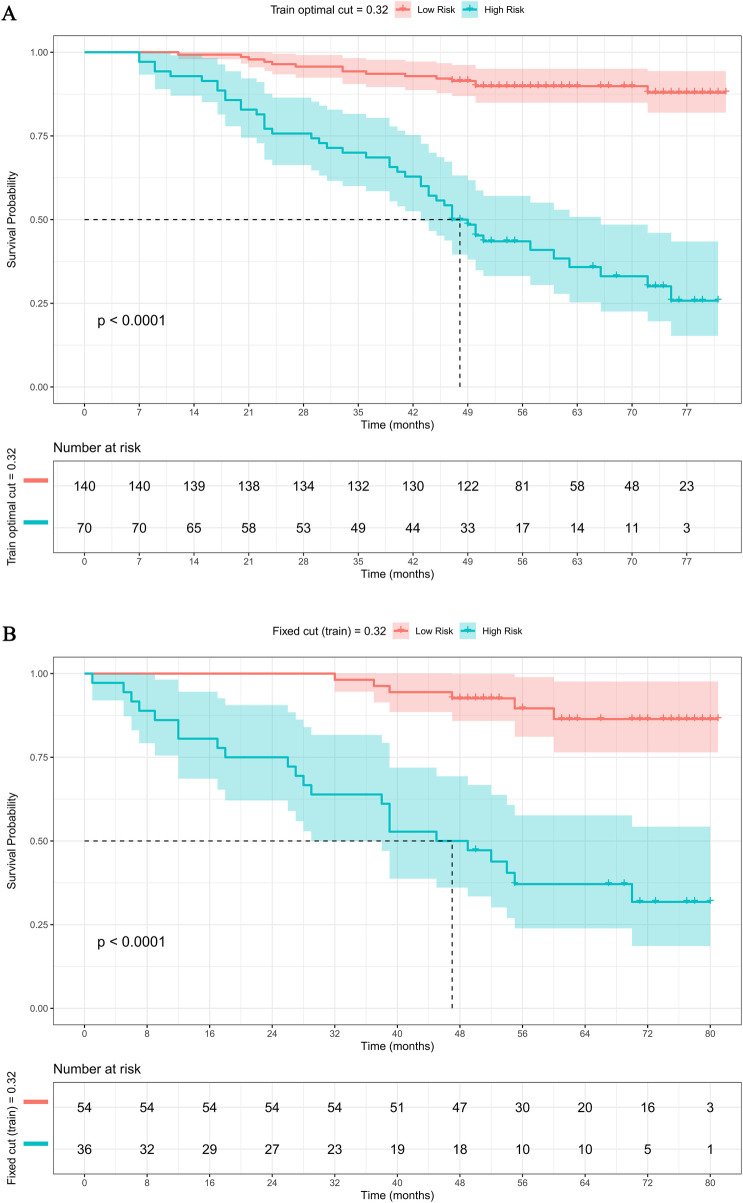
Survival analysis based on risk stratification by the nomogram. **(A)** Training cohort; **(B)** Validation cohort.

Using the linear predictor (LP) from the nomogram, an optimal risk-stratification cutoff of 0.32 was determined in the training set. This cutoff divided the training cohort into a low-risk group (*n* = 140) and a high-risk group (*n* = 70). Kaplan-Meier analysis showed significantly worse survival in the high-risk group [Log-rank *P* < 0.0001; Figure 6**:** (A) Training cohort]. When applied to the validation set, the same cutoff stratified patients into low-risk (*n* = 54) and high-risk (*n* = 36) groups, which again exhibited markedly separated survival curves [Log-rank *P* < 0.0001; Figure 6**:**(B) Validation cohort]. These consistent findings demonstrate the model's robust and effective capacity for risk stratification.

## 4 Discussion

Gastric cancer is a major global health challenge, ranking as the fifth most common cancer and the fourth leading cause of cancer death worldwide; it is the third leading cause of cancer mortality in China (14). Despite continual advancements in multidisciplinary treatment centered on surgery, postoperative outcomes show marked heterogeneity. Therefore, precise risk stratification is imperative to guide personalized treatment and follow-up. Currently, conventional serum tumor markers such as CEA and CA19-9 have limited sensitivity and specificity for prognosticating gastric cancer and fail to fully capture tumor biology or the patient's systemic status (15). This underscores the need for novel, clinically accessible prognostic indicators that can integrate multidimensional information.

Tumor-associated inflammation and the host's nutritional-immune status are increasingly recognized as key determinants of cancer pathogenesis, progression, and prognosis (6, 16). Systemic inflammation plays a dual role: it directly shapes the tumor microenvironment and fuels progression by suppressing anti-tumor immunity and promoting angiogenesis (17, 18). Concurrently, cancer-associated nutritional depletion and metabolic dysregulation impair treatment response and compromise long-term survival (19). Consequently, integrating inflammatory and nutritional markers into a composite assessment offers a promising strategy to more comprehensively evaluate a patient's pathophysiological state and obtain refined prognostic information.

The inflammatory burden index (IBI) and monocyte-to-albumin ratio (MAR) were selected for this study because they capture distinct but complementary aspects of the host-tumor relationship. IBI integrates C-reactive protein (CRP), neutrophils, and lymphocytes to reflect the balance between systemic inflammation and anti-tumor immunity. Elevated CRP and neutrophils typically indicate a pro-tumorigenic inflammatory state, which can promote angiogenesis and metastasis, while a relative lymphopenia suggests a compromised immune surveillance capability (20–23). By combining these parameters, IBI serves as a robust indicator of the “inflammatory-immune” axis, offering greater prognostic stability than single markers alone.

Similarly, MAR functions as a dual indicator of “inflammatory activation” and “nutritional depletion”. Monocytes are key progenitors of tumor-associated macrophages (TAMs), which facilitate tumor progression and immune evasion in the microenvironment (24). Serum albumin, conversely, reflects the host's nutritional reserve and metabolic resilience (25, 26). A high MAR therefore identifies patients who are simultaneously experiencing chronic inflammation and nutritional decline, a profile closely linked to poor postoperative recovery and survival.

The prognostic value of IBI and MAR has been demonstrated in hepatocellular carcinoma [Song et al. (8)] and non-small cell lung cancer [Zhao et al. (10)], respectively. In our cohort, IBI and MAR also emerged as independent predictors of overall survival. Using the optimal cut-off values, we further analyzed the associations of preoperative IBI and MAR with clinicopathological features. Both indices were significantly correlated with a more aggressive tumor phenotype, including poorer differentiation, larger tumor long diameters, advanced T and N stages, higher rates of vascular invasion, elevated CA19-9, and increased Ki-67 expression. Thus, these indices provide insight into both malignant tumor behavior and the host's systemic condition, thereby establishing their validity as prognostic biomarkers.

Multivariate Cox analysis identified preoperative IBI and MAR (as continuous variables), along with adjuvant therapy, N stage, and CA19-9, as independent predictors of overall survival after radical gastrectomy (all *P* < 0.05). Specifically, the N stage reflects the extent of lymph node metastasis and tumor invasiveness; a higher burden of metastatic nodes indicates stronger metastatic potential and correlates with increased recurrence risk, making it a cornerstone for prognostic stratification (27, 28). CA19-9, a specific marker for gastrointestinal malignancies, links closely to tumor cell proliferation and invasion. Elevated preoperative levels often signal a higher recurrence rate and shorter survival, while also serving as a metric for treatment response (29, 30). Conversely, adjuvant therapy acts as a protective factor by eliminating residual micrometastases (31). For patients with lymph node involvement or deep invasion, systemic chemotherapy effectively suppresses potential metastatic spread, significantly prolonging survival (32).

We integrated these independent predictors to develop a prognostic nomogram, which was then subjected to internal validation. The model demonstrated excellent predictive performance, with concordance indices (C-index) of 0.809 in the training set and 0.802 in the validation set. The time-dependent AUCs for 1-, 3-, and 5-year overall survival all exceeded 0.83. Furthermore, calibration curves showed close agreement between predicted and observed survival. These results collectively demonstrate that the nomogram possesses strong discrimination and calibration. Importantly, the nomogram allowed for clear stratification into low- and high-risk groups based on its linear predictor. This stratification was highly reproducible, with significantly separated survival curves in both the training and validation sets (Log-rank *P* < 0.0001). The clinical utility of this tool is substantial, as it facilitates the rapid identification of high-risk patients, thereby guiding the formulation of intensified follow-up regimens and adjuvant strategies. Specifically, for high-risk individuals, increasing the frequency of imaging and tumor marker monitoring can allow for the early detection of recurrence and timely intervention. Beyond clinical decision-making, the visual nature of the nomogram also enhances doctor-patient communication, enabling patients to intuitively understand their prognostic outlook. It is important to note that while IBI, MAR, and CA19-9 are typically measured preoperatively, this nomogram integrates them with postoperative pathological findings (N stage) and treatment factors (adjuvant therapy). Therefore, this model is specifically designed to be applied in the postoperative setting—typically after the completion of pathological staging and the determination of the adjuvant therapy plan—to predict long-term survival.

This study has several limitations. First, its single-center, retrospective design may introduce selection bias. Second, the lack of an independent external cohort limits the generalizability of the findings. Consequently, this nomogram should currently be considered a proof-of-concept model and is not yet ready for routine clinical use. Therefore, the results must be interpreted with caution. To address this, we plan to conduct future multicenter, prospective studies to provide robust external validation of the risk-score cutoff and the model's predictive performance.

## 5 Conclusion

In summary, this retrospective study confirmed that preoperative inflammatory burden index (IBI) and monocyte-to-albumin ratio (MAR) are independent prognostic factors for overall survival after radical gastrectomy. By integrating these markers with standard clinicopathological parameters, we developed a nomogram that shows excellent discrimination and calibration. However, given the lack of external validation, this tool should currently be considered a proof-of-concept. Pending further validation, it holds potential as a visual aid for individual postoperative prognosis assessment and risk-based management.

## Data Availability

The raw data supporting the conclusions of this article will be made available by the authors, without undue reservation.
